# Antistreptococcic Serum

**Published:** 1905-01-07

**Authors:** 


### ANTISTREPTOCOCCIC SERUM.

There can be no doubt whatever that antistreptococcus serum is a therapeutic agent which has come to stay and which will find a wider field of usefulness as our knowledge of micro-organisms grows and our methods of preparing the serum are correspondingly improved. All that can be said with regard to the success attending its use up to the present is that the numerous instances in which the serum has failed to effect any material result on the morbid process are offset by an occasional case in which a brilliant success has been attained and a life saved by the timely injection of antistreptococcus serum. It is becoming increasingly evident that the uncertain results of the past are to be attributed to a want of discrimination of the different kinds of streptococci. A distinct advance was made when certain manufacturing firms prepared immunising serums by using several different strains of streptococci from various sources; but there is much that still remains to be done in amplification of this principle. In last week's number of the *Lancet* there is an article written by Mr. A. G. R. Foulerton in which he describes some work which he has carried out with Dr. Victor Bonney along these lines. They examined bacteriologically the contents of the uterus in 54 cases of fever following on either miscarriage or labour at full term. In 46·3 per cent. of these cases they found streptococci under conditions which indicated that they were the primary cause of the fever. Excluding the less severe cases, and considering only those which ended in death or were clinically of a distinctly serious nature, they found streptococci present in 62·5 per cent.

From these cases 25 strains of streptococci were obtained and studied, and then an anti-streptococcus serum was obtained by vaccinating a horse with five of the strains. The serum obtained in this way has already been used in a few cases with considerable success; and Dr. Foulerton remarks that, while the special value of a serum thus prepared for use in puerperal cases as against the ordinary polyvalent antistreptococcus serum hitherto used has yet to be proved, the opinion is justified that if the serum treatment of streptococcic puerperal fever is to be placed on a satisfactory basis, it must be by means of some such method as that which he and Dr. Bonney have endeavoured to carry out. It is greatly to be desired that the hint will fall upon appreciative ears, and that the preparation of polyvalent anti-streptococcic sera will in the future be more scientifically performed, when we may also expect to find it more potent to cure.

